# Two-photon autofluorescence lifetime imaging of human skin papillary dermis *in vivo*: assessment of blood capillaries and structural proteins localization

**DOI:** 10.1038/s41598-017-01238-w

**Published:** 2017-04-26

**Authors:** Evgeny A. Shirshin, Yury I. Gurfinkel, Alexander V. Priezzhev, Victor V. Fadeev, Juergen Lademann, Maxim E. Darvin

**Affiliations:** 1 Faculty of Physics, Lomonosov Moscow State University, Moscow, Russia; 2 Research Clinical Center of JSC “Russian Railways”, Moscow, Russia; 3 Department of Dermatology, Venerology and Allergology, Center of Experimental and Applied Cutaneous Physiology, Charité –Universitätsmedizin Berlin, Berlin, Germany

## Abstract

The papillary dermis of human skin is responsible for its biomechanical properties and for supply of epidermis with chemicals. Dermis is mainly composed of structural protein molecules, including collagen and elastin, and contains blood capillaries. Connective tissue diseases, as well as cardiovascular complications have manifestations on the molecular level in the papillary dermis (e.g. alteration of collagen I and III content) and in the capillary structure. In this paper we assessed the molecular structure of internal and external regions of skin capillaries using two-photon fluorescence lifetime imaging (FLIM) of endogenous compounds. It was shown that the capillaries are characterized by a fast fluorescence decay, which is originated from red blood cells and blood plasma. Using the second harmonic generation signal, FLIM segmentation was performed, which provided for spatial localization and fluorescence decay parameters distribution of collagen I and elastin in the dermal papillae. It was demonstrated that the lifetime distribution was different for the inner area of dermal papillae around the capillary loop that was suggested to be due to collagen III. Hence, we propose a generalized approach to two-photon imaging of the papillary dermis components, which extends the capabilities of this technique in skin diagnosis.

## Introduction

Skin is the largest organ in the human body serving numerous functions^1^. Generally, skin consists of three distinct layers – epidermis, dermis and the inner layer – hypodermis, which mainly contains fat cells, adipocytes. Epidermis is composed of cell layers and is attached to an underlying dermis with a basement membrane (BM), which controls an exchange of chemicals between blood vessels and epidermis. The BM structure is determined by the interaction of epidermal cells, keratinocytes, and fibroblasts from the papillary dermis, which produce necessary protein components, including collagen type IV^1–4^.

The dermis consists of two layers – papillary (*stratum papillare*) and reticular dermis (*stratum reticulare*). The depth of a papillary dermis is approximately 300–400 μm, and its upper part is arranged into ridge-like structures, the dermal papillae, which contain microvascular and neural structures^1^. Dermal papillae greatly extend the surface area of the dermal-junction and facilitate delivery of soluble molecules to the epidermis from blood capillaries. A typical adult has about 10^11^ blood vessels, and more than 99% of these vessels are involved in the microcirculation – a vast network of interconnected vessels^5^. This network is controlled by numerous mechanisms, and there are many ways in which such a delicate system may break down, leading to cardiovascular disease. The structure of capillaries can also be influenced by dermal diseases such as psoriasis^6–8^. Skin capillaries can be observed using standard optical microscopy^9^. In the common case, the superficial capillary loops are oriented perpendicular to skin surface, hence, only their terminal parts can be distinguished. However, in the fingernail bed area capillaries are aligned parallel to the surface, and can be inspected across the whole length^9^. Digital optical capillaroscopy provides clinically important information on microvascular abnormalities in patients. For instance, the density of the capillary network and the ratio of the capillaries’ venous segment to arterial segment diameters could give quantitative evidence for characterization of arterial hypertension stages^10^. Though being informative in terms of prediction of microvascular complications, whose earliest manifestations are encountered in the microcirculation, as well as other pathologies^11^, simple visualization of capillaries with optical microscopy does not allow for assessing of molecular species and biochemical processes in blood. At the same time, *in vivo* blood cytometry, which can be performed through skin noninvasively, is highly desirable for biomedical diagnosis and can be potentially performed by complex methods^12, 13^.

Dermis is mainly composed of extracellular matrix (ECM) produced by fibroblasts^3^, with collagen being the most abundant protein component. Different subpopulations of fibroblasts result in differences between the ECM organization in different areas of dermis. At that, papillary dermis is characterized by thin, randomly oriented fibers made of collagen types I and III, intercrossed with the elastin fibers, while reticular dermis is made of thin collagen fibers^1^. The ratio and the interaction between collagen type I and type III determine biomechanical properties of the connective tissue^4^. Various diseases, as well as connective tissue disorders, have manifestations at the level of dermis. Keloid, morphea, dermal elastosis, skin aging and photodamage are associated with reorganization of the ECM and rearrangement of collagen fibers’ architecture and content^14–17^. Collagen remodeling is also a central process during wound healing^18, 19^, and its monitoring *in vivo* is important for regenerative medicine^20^. Current techniques to visualize and quantify changes in tissue collagen types rely on immunohistochemistry and polarized staining under the microscope^21, 22^. Although histopathology is the golden standard and provides for the highest accuracy and specificity in detection of tissue pathologies, it requires time-consuming tissue processing and invasive procedures, which is not suitable e.g. for a routine investigation of cosmetic problems. This led to an impetuous development of several *in vivo* methods, which allow for the assessment of skin structure, including high frequency ultrasonography^23^, optical coherence tomography (OCT)^24, 25^, confocal laser scanning microscopy (LSM)^26–28^, confocal Raman microscopy (RM)^29, 30^ and multiphoton imaging^31^, including multiphoton tomography (MPT)^32^ combined with coherent anti-Stokes Raman scattering (CARS)^33, 34^.

MPT is based on non-linear optical effects such as higher optical harmonics generation (e.g., SHG, Second Harmonics Generation), two photon excited autofluorescence (TPEAF) and CARS, which require the use of short (usually femtosecond) excitation pulses. MPT allows for non-invasive imaging of tissue structure at the subcellular level with a real-time temporal resolution^32^. The major advantage of this technique is the possibility of assessment of biochemical properties of tissues, i.e. molecular composition at certain spatial points, especially when MPT is combined with time-resolved methods, i.e. pump-and-probe spectroscopy and FLIM (Fluorescence Lifetime Imaging)^32, 35^.

MPT allows for the evaluation of tissue architecture and molecular composition in the upper dermis. While the SHG signal in the dermis mainly originates from collagen I fibers and can be used for their quantification and morphology assessment^36^, intense TPEAF from this skin region is considered to be due to elastin fibers^37^. As a result, several descriptors have been developed to assess the morphological state of the upper dermis on the basis of MPT^37–39^, thus solving the principal quantification problem for dermatology and cosmetology. It was also shown that SHG microscopy itself is a powerful method, which allows obtaining detailed information on the collagen I fibers structure and their arrangement in tissues, providing for valuable clinical information^40–43^. However, signal separation for different collagen types is not readily provided by the MPT.

Though fluorescent properties of different collagen types in model systems have been studied^44–46^, their separation in fluorescence images for real objects, especially *in vivo*, still remains a challenging problem and requires an application of FLIM with advanced processing algorithms^47^, which in the case of dermis requires separation of autofluorescence of other ECM components, e.g. elastin. Fluorescence lifetimes of elastin and collagen have been multiply addressed in relation to measurements of aorta walls constituents^48, 49^. It was shown that the absolute lifetime values of these species vary significantly depending on the sample preparation, storage and environment, e.g. fluorescence lifetime for model preparations elastin differs from that for elastin in aorta^44, 48^. Generally, the data on the absolute lifetime values of collagen I and elastin is rather contradictive^44–49^, and implementation of an independent method, e.g. SHG, is required for reliable localization of collagen and elastin in tissues.

While a lot of works using MPT was performed on epidermis^50–53^, only a sparse information was reported on microvascular components in the papillary dermis. For instance, it is a common knowledge that the location of capillary in papillae is seen as a hollow area inside the collagen I matrix in the SHG signal^40, 42, 43^, however, applications of MPT in the dermal microvasculature research are almost lacking. In this work, we aimed at the investigation of capillaries in dermal papillae of human skin *in vivo* with a focus on the possibility of their imaging with MPT-FLIM using autofluorescence of the endogenous compounds. For this purpose, the origin of capillary-related fluorophores at two-photon excitation was investigated. We also performed a detailed investigation of the molecular species localization in papillary dermis around the capillaries using segmentation of FLIM images guided by the intensity distributions of SHG and TPEAF. The obtained results suggest the existence of at least three distinct ECM components in the dermal papillae, two of them being collagen I and elastin, and the third, presumably, collagen III. Finally, we propose a generalized approach to MPT imaging of capillaries interior, including blood plasma, and neighboring tissues in the framework, which extends the standard collagen-elastin picture of dermis composition accepted in MPT studies.

## Results and Discussion

### LSM imaging of capillaries in papillae

The major aim of the present research was to assess the molecular structure of internal and external regions for skin capillaries with two-photon imaging, but previously to that characterization of the corresponding structures with LSM operated in the reflection mode^54^ was performed.

**Figure 1:**
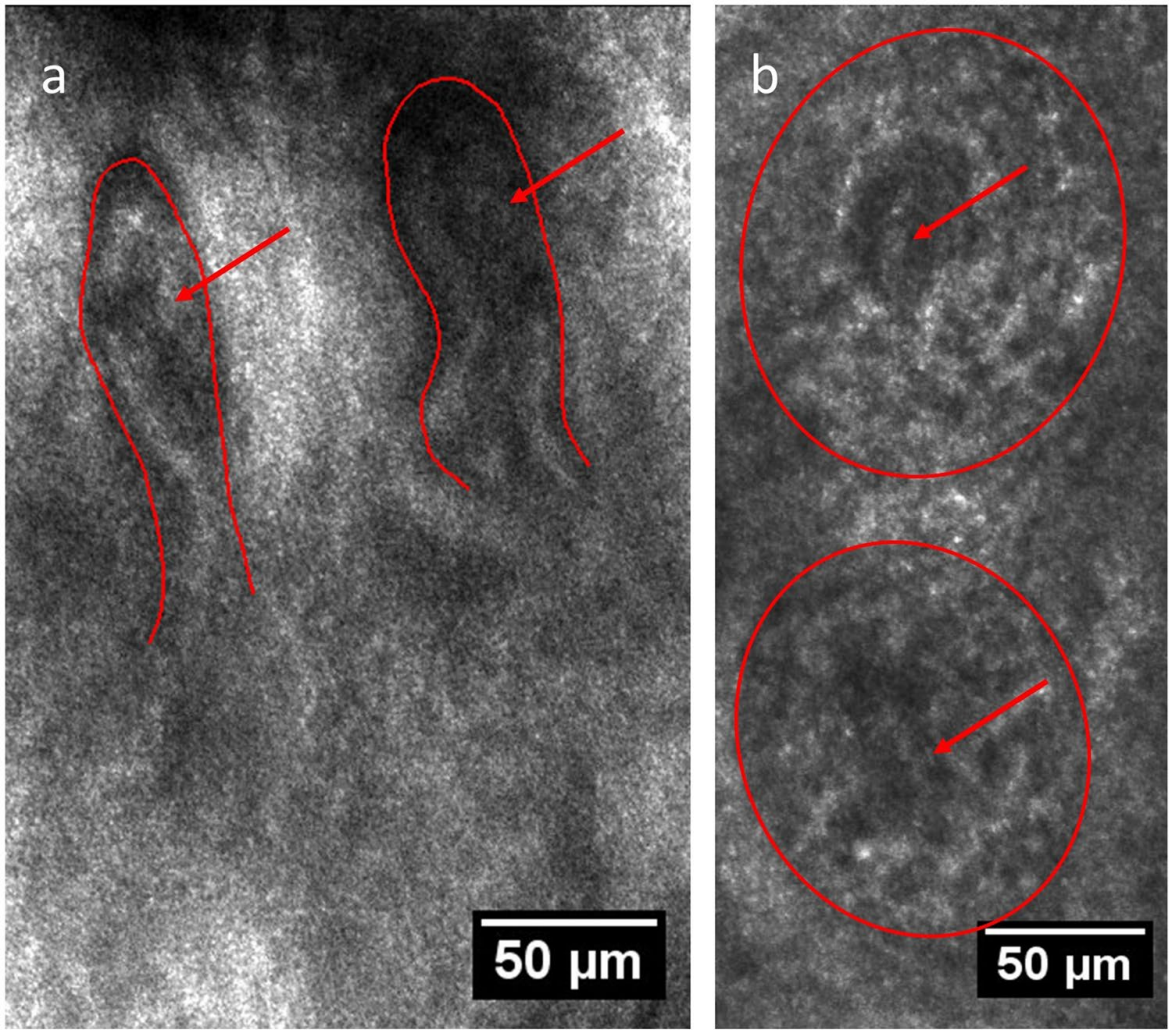
Capillaries of human skin *in vivo* in (**a**) fingernail bed, 150 μm depth, (**b**) inner forearm, 60 μm depth as seen via LSM (reflection mode); the red lines correspond to papillae borders, and the red arrows indicate the position of capillary loops inside the papillae. The scanning wavelength was set to 785 nm.

Figure 1a demonstrates the image of nailbed capillaries obtained using LSM in a reflectance mode under 785 nm light excitation. Pronounced structure of capillaries in fingernail bed makes them a convenient object for visualization and quantification of the microvascular network. In contrast to that, papillary capillaries in other regions of skin are oriented perpendicular to the surface. The capillary loops in the inner forearm at ~60 μm depth can be seen in Fig. 1b. Importantly, the blood flow was clearly seen in the LSM video mode both for the fingernail and forearm capillaries (see the Videos 1 and 2).

Using LSM (fluorescence mode) on the fingernail bed region, no fluorescence was observed from the papillary area upon excitation at 488 nm, which could be due to the strong light absorbance and scattering in tissue, as well as due to the absence of a sufficient number of fluorophores excited by this wavelength.

### Two-photon imaging of fingernail bed capillary loops

As the fingernail bed capillaries exhibit a pronounced horizontally oriented structure, which can be easily recognized, we started our experiments on two-photon imaging of blood vessels from the fingernail bed area.

**Figure 2:**
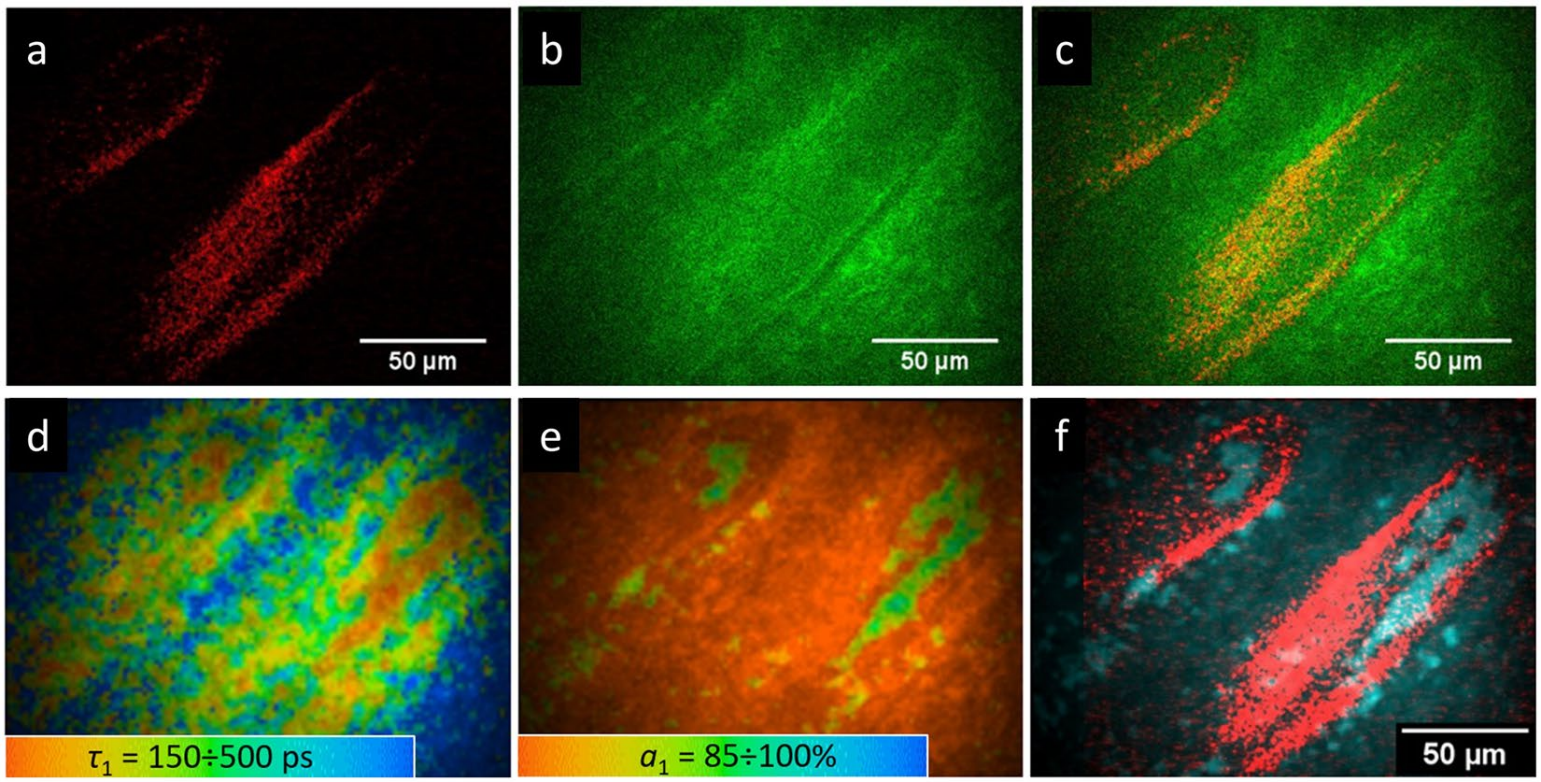
Two-photon imaging of the fingernail bed area: (**a**) SHG signal, (**b**) TPEAF signal, (**c**) merged SHG and TPEAF signals, (**d**) FLIM image, colors correspond to the *τ*_1_ values (the color scale corresponds to the 150–500 ps range), (**e**) FLIM image, colors correspond to the a_1_ values (the color scale corresponds to the 85–100% range), (**f**) merged SHG and FLIM images.

In the dermis (at ~150 μm depth) prolonged finger-like structures could be observed in the SHG channel (Fig. 2a), suggesting that these are horizontally aligned papillae where the capillaries should reside.

The SHG signal in human skin originates from collagen I fibers, which are characterized by a needle-like non-centrosymmetric geometry and hyper polarizability. They give rise to a non-zero second order susceptibility. This approach is widely used to study the organization of collagen fibers in the human dermis^55^. TPEAF from the same area, which is observed simultaneously with the SHG signal, is presented in Fig. 2b. Figure 2c shows the false-color image of the merged SHG and TPEAF signals, demonstrating the orientation of papillae in the fingernail bed. As can be seen, no clear signs of the capillary can be observed in this image.

In contrast to this, the FLIM images, that represent the distribution of fluorescence decay parameters over the selected area, demonstrate the structure, which can be readily attributed to the capillary. This is confirmed by the capillary geometry obtained using LSM. At that, the colors in Fig. 2d correspond to the values of the fast component in fluorescence decay (*τ*_1_), and the colors in Fig. 2e correspond to the values of amplitude *a*_1_ of this fast component in fluorescence decay. The capillary-like structure is characterized by a fast fluorescence decay (*τ*_1_ ~ 150 ps) and high amplitude of the fast component (*a*_1_ ~ 90%). Figure 2f demonstrates the merged image of the SHG signal (red) and signal with fast fluorescence decay (*τ*_1_ < 200 ps, *a*_1_ > 80%), illustrating that the hollow areas in the papillae fit the capillary structure. We also observed that upon a tense pressure on the fingernail bed during measurements the capillary-induced signal in FLIM disappeared, suggesting that the fast fluorescence decay is due to the capillary’s content, i.e. blood, but not due to the capillary walls. To further investigate the origin of the fluorescence signal from the area of the capillary, we performed a separate study of two-photon fluorescence of blood, namely, of its two main components – red blood cells (RBCs) and blood plasma.

### TPEAF/FLIM of human red blood cells and blood plasma

**Figure 3:**
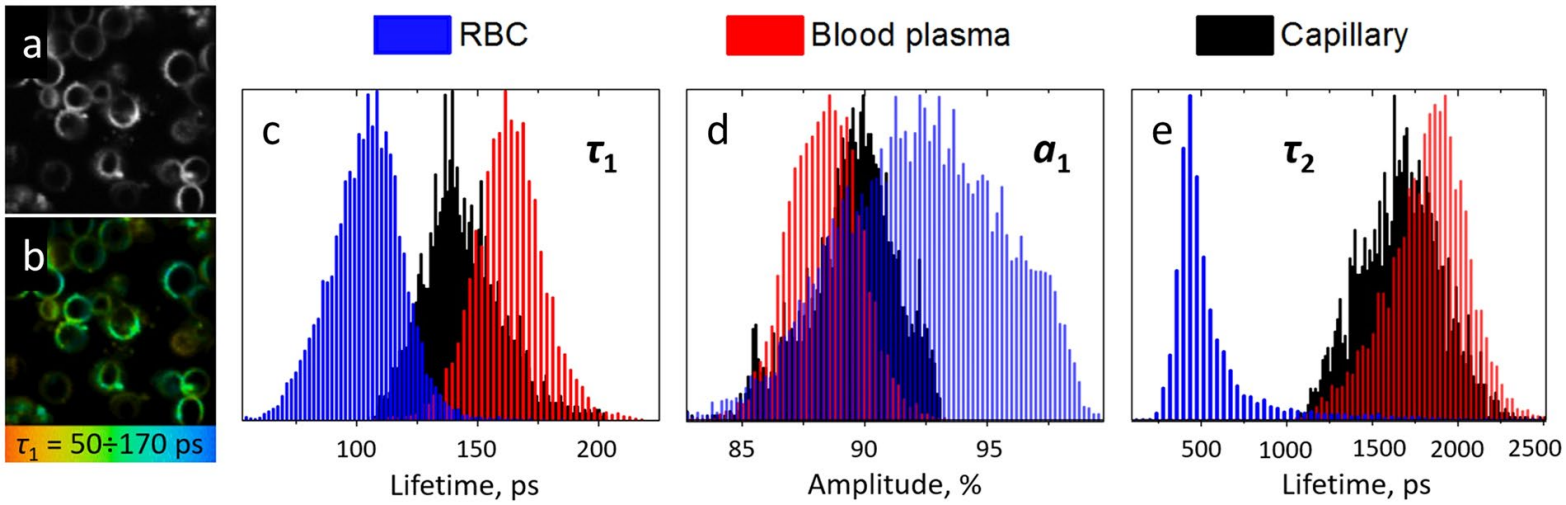
(**a**) TPEAF and (**b**) FLIM images of RBCs obtained at 760 nm excitation (2 mW on the sample, the image width is 50 µm). The colors in Fig. 3b correspond to the 50–170 ps range for τ_1_, (**c**–**e**) Normalized distributions of the *τ*_1_, *a*_1_ and *τ*_2_ parameters obtained for RBCs (blue), blood plasma (red) and capillary (black). The FLIM images were fitted biexponentially for the pixels with maximum intensity exceeding 200 counts.

To verify the origin of fluorescence located in the capillaries area we performed the measurements of human RBCs and blood plasma. It has been reported that hemoglobin exhibits high two-photon absorption cross-section, and that two-photon excitation of hemoglobin results in a high energy Soret fluorescence with a fast decay^56–58^. TPEAF of hemoglobin, which is the major intracellular component of RBCs with the concentration of ~300 mg/ml, makes it a prominent marker for non-invasive imaging of blood vessels^57, 58^. Hence, TPEAF fluorescence of hemoglobin could be considered as a major component responsible for blood vessels signature in the FLIM signal. Indeed, the results of RBC’s TPEAF measurements show that RBCs are characterized by a fast fluorescence decay with a narrow lifetime distribution (100 ± 20 ps) and high impact of the fast component’s amplitude a_1_ (Fig. 3). The slower decay component observed for RBCs was about 500 ps and could be probably originated from NAD(P)H^59^.

We also investigated TPEAF of the second major component of human blood – blood plasma. Upon excitation of a blood plasma liquid drop with 760 nm laser pulses a pronounced fluorescence signal was observed, the intensity of which was several times lower compared to RBCs. The fluorescence decay parameters for blood plasma are presented in Fig. 3, which are characterized by a quite narrow distribution of τ_1_ (170 ± 20 ps), that is characteristic of single fluorophore predominance in the overall fluorescence signal, and high a_1_/a_2_ ratio, which was comparable to that of hemoglobin. The slow component τ_2_ with 1–2 ns lifetime, a broad distribution (FWHM ~ 500 ns) and ~10% amplitude in the fluorescence decay could be supposedly due to the fluorescence decay artefacts. However, during the processing of blood plasma FLIM images we took into account only bright pixels with >500 counts intensity in maximum, hence, we expect this decay component to be originated from real fluorophores. Though the origin of these fluorophores requires further investigation, we note that at one-photon excitation in this wavelength region (~380 nm) numerous fluorophores are known to emit fluorescence in blood plasma, such as different advanced glycation end-products, NAD(P)H, fluorescent protein cross-links, etc.^60, 61^. However, the fast component of blood plasma decay (200 ± 20 ps) is sufficiently lower than that for NAD(P)H (~500 ps), which is known to contribute significantly to the blood plasma emission^62^. Fluorescence of biological liquids, especially of blood plasma, is extensively studied, that is motivated by the necessity to develop novel methods for the detection of pathologies and metabolic disorders in the human organism^60–62^. Collectively, our results suggest that TPEAF could be a prospective method to assess the fluorescence of blood plasma *in vivo*.

Figures 3c–e also demonstrate the fluorescence decay parameters distribution obtained for the capillary area presented in Fig. 2d–f. It can be seen that the τ_1_ and a_1_ values for capillary are somewhere in between the corresponding parameters obtained for RBC and blood plasma, as one could expect.

We note that in our experiments a single FLIM image with a 300 µm width was measured during 6.2 s, which correspond to the 50 µm/s scan rate. Considering the blood flow velocity in capillaries of ~500 µm/s^63^, one could expect a homogeneous distribution of fluorophores in the measurement area, i.e., no single RBCs could be observed and the resulting signal would be a mixture of RBCs and blood plasma fluorescence with a predominant impact of RBCs. However, the longer lifetime component (τ_2_ ~1.7 ns) present in the capillaries could be associated with blood plasma fluorescence as it is completely lacking in the RBC-induced FLIM signal (Fig. 3e).

### FLIM of the forearm capillaries

**Figure 4:**
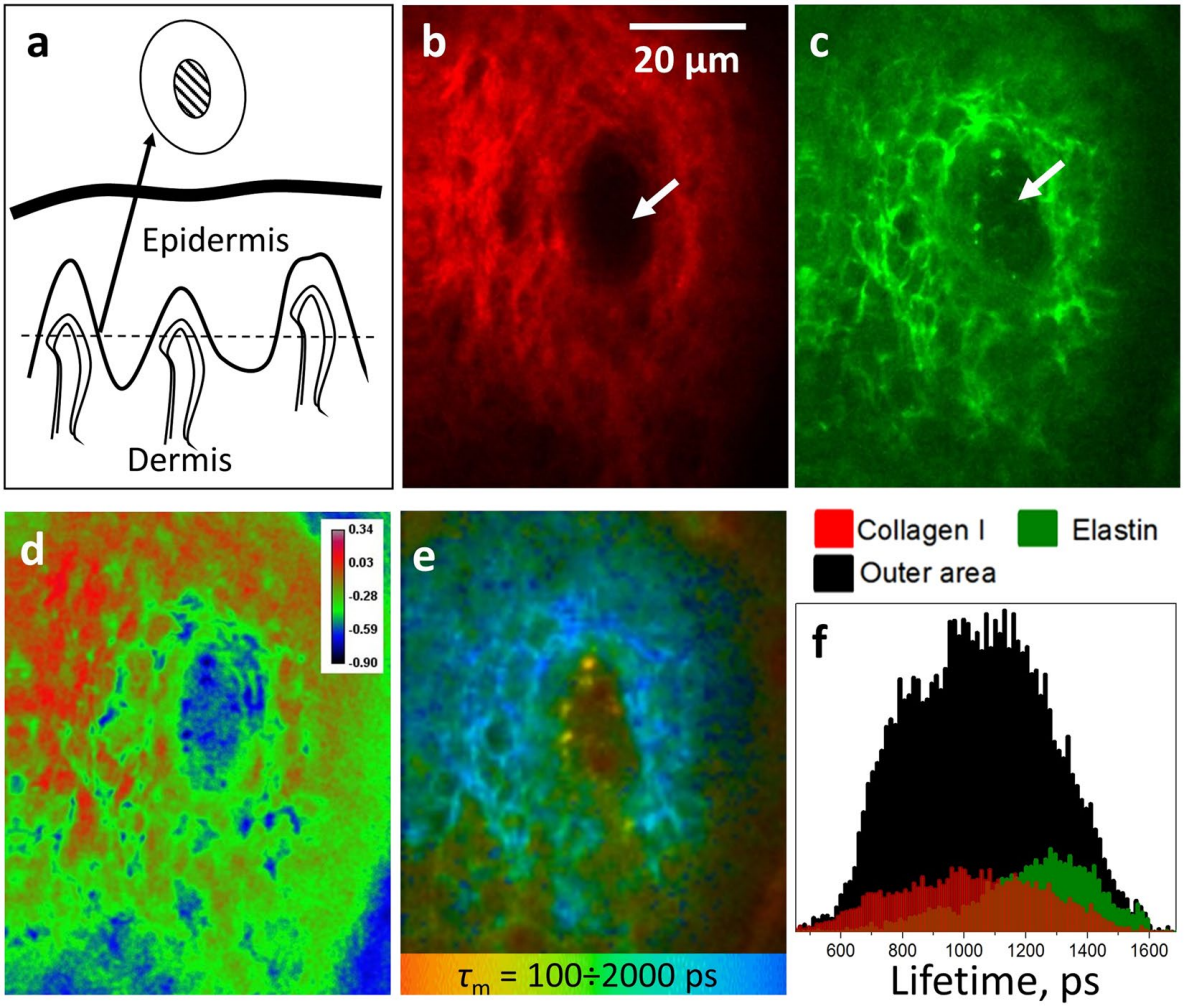
(**a**) Schematic representation of the skin structure. The dashed line corresponds to the plane of the papillary dermis cross-section measured with MPT, the outer and inner circles correspond to the cross-section of dermal papilla and the inner area around capillary. (**b**,**c**) Two-photon images of the dermal papilla: SHG and TPAEF signals, respectively. The white arrows correspond to the “hollow” area inside the papilla. (**d**) Spatial distribution of the SAAID index (SAAID = (SHG – TPEAF)/(SHG + TPEAF)) in the dermal papilla. (**e**) FLIM image of the dermal papilla, pixel colors correspond to different values of the mean fluorescence lifetime *τ*_m_ in the 100 ÷ 2000 ps range. (**f**) Mean lifetime distributions obtained for the outer area, positive SAAID (>0.15, collagen I) and negative SAAID (−0.4 ÷ −0.35) regions of the dermal papilla (see text for details).

In contrast to the fingernail bed, the capillaries in the inner forearm are oriented perpendicular to skin surface, and only a transverse cross-section of U-shaped capillary loops can be obtained (Fig. 4a). Figure 4b,c demonstrate representative two-photon images taken at 60 µm depth in the inner forearm of a healthy individual, which show the following features. Collagen I fibers of the papillae can be observed in the SHG channel (Fig. 4b), while fibrous structure revealed in TPEAF could be attributed to elastin (Fig. 4c). In Fig. 4b,c a hollow area can be observed in the middle of the papillary structure (white arrow), where no SHG and weak TPEAF signals are observed. Figure 4d demonstrates the spatial distribution of the SAAID (SHG to Autofluorescence Aging Index of Dermis) index across the papilla, which is characteristic of the collagen-to-elastin ratio in the skin^37, 38^. Positive SAAID values (red color) correspond to collagen I predominance, and negative SAAID values (green color) correspond to the elastin-rich areas.

FLIM data demonstrates much more detailed aspects of the dermal papilla organization (Fig. 4e, colors correspond to different values of *τ*_m_), and their analysis allows for the following localization of fluorescing species. First, fast fluorescence decay (red color in Fig. 4e) can be observed for two spots in the center of the papilla, which represent the cross-section of the U-shaped capillary loop. Indeed, a capillary must be present inside the papilla, and fluorescence properties of these two spots coincide with that of capillaries from the fingernail bed (Fig. 2d). The FLIM pictures taken at different depths are presented in the Video 3, where the consequent cross-sections of the U-shaped capillary loops can be clearly observed. The fluorescence contour around the papilla with characteristic fast decay (Fig. 4e) could be attributed to melanin-containing cells^52^. We note that visualization of the *rete ridges* by separating fast melanin fluorescence in FLIM (blue color in the Video 3) could be used to reconstruct the morphology of the dermal-epidermal junction, which is indicative of skin pathologies^64^.

Second, bright fibrous regions in the TPEAF image (Fig. 4c) correspond to the light-blue areas of the FLIM image (Fig. 4e). These fragments could be associated with elastin fibers in papillary dermis. Third, the green regions inside the papilla outside the “hollow” area in Fig. 4e correspond to the red regions in Fig. 4d, i.e. to the positive SAAID. These regions are rich in collagen I and exhibit lower fluorescence compared to bright blue areas in Fig. 4d. Off note, yellow areas with the average lifetime of 500 ± 100 ps and the average size of 6 ± 2 μm were present in more than 30% of the obtained dermal papillae FLIM images in their inner parts. Though the origin of the observed areas requires further investigation, we suppose that they could be originated from cell components, which could be present near the capillary in the perivascular space^65^.

Finally, the green area, which surrounds blood vessels in the middle of papilla and corresponds to the “hollow” area (white arrows in Fig. 4) can be clearly observed in the FLIM image (Fig. 4e). As no SHG is detected for this region, this signal couldn’t have been caused by collagen I. At the same time, elastin-rich regions are colored in blue in Fig. 4e, as described above. These facts suggest that the inner (“hollow”) area of the papillary structure, which surrounds the capillary loop, predominantly contains molecular species different from collagen I and elastin.

Using the SAAID value for different parts of the papilla (Fig. 4d), fluorescence lifetime distributions can be obtained for its outer area and collagen I-rich (positive SAAID, high SHG signal) and elastin-rich (negative SAAID, high TPAEF intensity) regions as shown in Fig. 4f. It can be seen that the mean fluorescence lifetimes for collagen I and elastin represent the fast and slow parts in the overall distribution obtained for the outer area of the papilla. However, the presence of other fluorescing species in the outer area of the papilla can’t be excluded.

**Figure 5:**
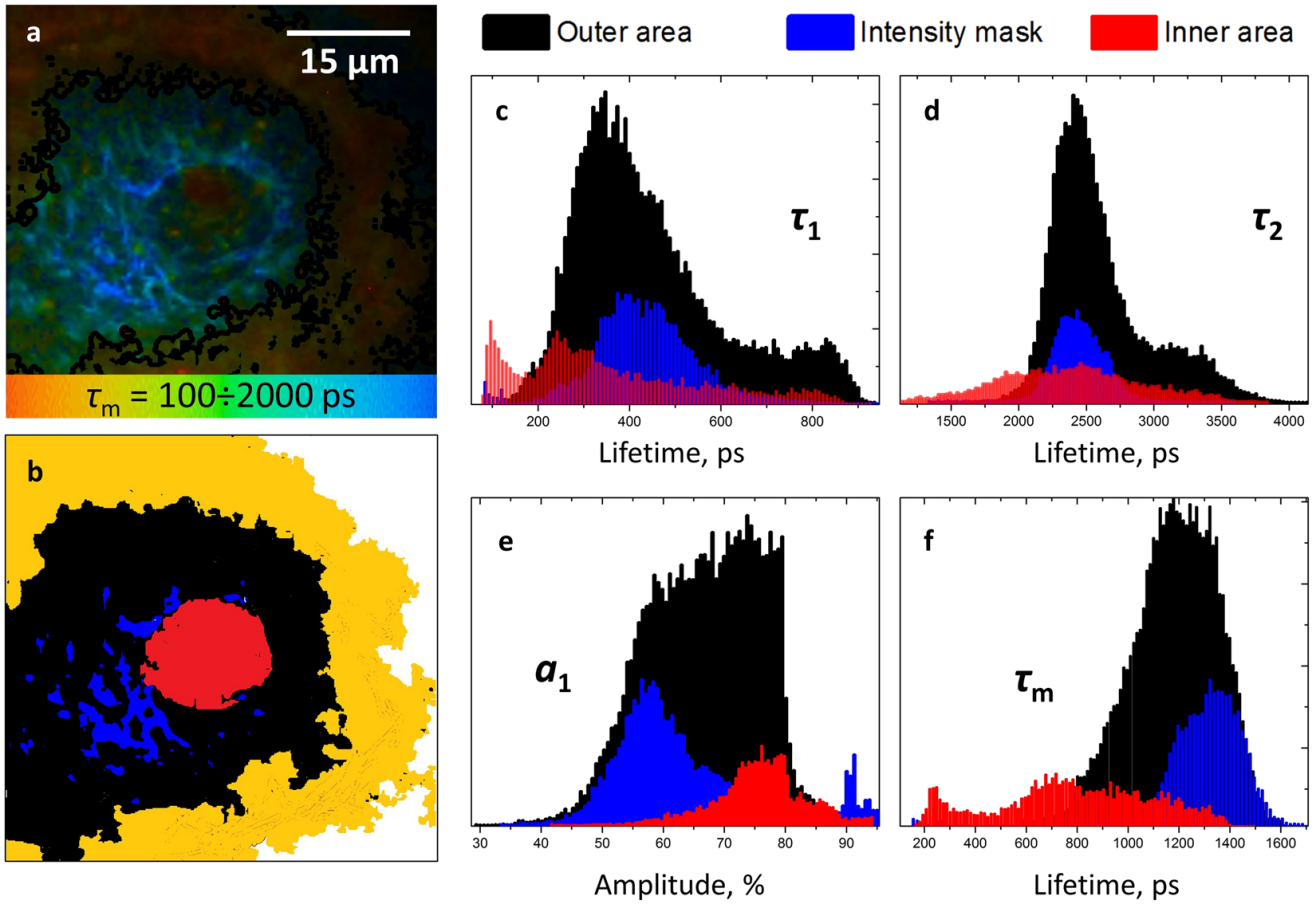
(**a**) FLIM image of the papillary structure, colors correspond to the values of mean fluorescence lifetime in the 100 ÷ 2000 ps range. (**b**) segmentation of the FLIM image of papilla into the inner area (red), outer area (black) and intensity mask area (blue). The yellow area corresponds to the melanin-rich area around the papilla. (**c**–**f**) – distributions of fast (*τ*_1_) and slow (*τ*_2_) decay components, amplitude of the fast component *a*_1_ and mean fluorescence lifetime, respectively.

A more detailed analysis of fluorescence decay parameters is presented in Fig. 5. The dermal papilla was segmented into three regions – the inner and outer areas, colored in red and black, respectively, and the “intensity mask” area, which corresponds to pixels in the outer area with the highest autofluorescence intensity (Fig. 5a,b). The cutoff value for the intensity mask (15% of the brightest pixels) was taken arbitrary to demonstrate the origin of bright fibrous structures inside the papilla – as this regions were characterized by the slowest decay, this area could be expected to be elastin-rich, and the lifetime distribution for this area should be similar to the distribution for elastin shown in Fig. 4f. The inner area was selected as the region inside the papilla where the SHG signal was absent (see also Fig. 4a,b).

The obtained lifetime and amplitude distributions for the selected areas are presented in Fig. 5c–f, and the corresponding values are summarized in Table 1. As expected, the lifetime distribution under the intensity mask (blue color in Fig. 5b) was close to the distribution for elastin fibers (Fig. 4f), and the distributions obtained for the outer area could be interpreted as previously, namely, as a sum of distributions for collagen and elastin.

**Table 1:** Fluorescence decay parameters obtained for the ECM components in the papillary dermis.

	*τ*_1_, ps	*τ*_2_, ps	*a*_1_/*a*_2_	*τ*_m_
Elastin	400 ± 70	2300 ± 200	1.2 ± 0.2	1300 ± 100
Collagen I	300 ± 50	2500 ± 200	1.8 ± 0.2	1000 ± 150
Inner area ECM	250 ± 70	2300 ± 300	3.0 ± 0.4	800 ± 150

The lifetime distributions were different for the inner area, confirming that the corresponding fluorophore(s), different from collagen I and elastin, yields the fast part of the *τ*_m_ distribution. For instance, the average lifetime *τ*_m_ distribution for the inner area is centered at ~800 ps, while *τ*_m_ for elastin is centered at ~1300 ps (Fig. 5f). At the same time, though *τ*_m_ for collagen I is shorter (~1000 ps), no collagen I could be expected in the inner area due to the lack of SHG signal.

### Protein species in the papillary dermis

Localization of the ECM components in the papillary dermis *in vivo* is connected with two tasks: (i) separation between collagen and elastin and (ii) separation of different types of collagen.

Multiphoton tomography is capable of providing for the ratios of collagen and elastin fractions in the dermis based on the suggestion that at a certain depth the SHG signal originates solely from collagen I, while the TPEAF is mainly due to elastin molecules^37, 38, 66^. The corresponding collagen index, SAAID, is almost constant with depth at z > 120 µm and can be used as an integral indicator of the skin state without providing for spatial localization of ECM components^66^.

Koehler *et al*.^67^ reported the differences in lifetime distributions for the dermis obtained using MPT depending on the patients’ age and skin area localization (sun-protected or sun-exposed), that was attributed to different content of collagen I and elastin. However, this research investigated neither spatial lifetime distribution nor attribution of lifetimes to certain fluorophores. The results of investigation of collagen and elastin components performed in different systems using time-resolved fluorescence spectroscopy^44, 48, 49^ suggest that this task can be solved by taking into account the relative differences between their lifetimes, but not their absolute values.

The collagen-to-elastin ratio has been also quantified using spectrally-resolved MPT without time resolution^68^. In this case the authors measured fluorescence spectra from each point of the MPT image and after that performed clustering of different regions based on a phasor plot in a spectral domain, which allowed to distinguish collagen-rich areas by specific SHG-related features. Interestingly, this work presented clustered images of papillary structures with melanin-containing cells around them, which contain characteristic “hollow” regions inside like the ones shown in Fig. 4, however, no blood vessels were identified using the proposed approach.

The major collagen constituents of the ECM in the papillary dermis are collagen types I and III, moreover, their ratio is of interest for numerous clinical applications, including the monitoring of the proliferation and remodeling phase of wound healing^18–20^. Collagens of different types and sources exhibit fluorescence emission which varies in spectral band shape, position of maximum and fluorescence lifetimes^44–47, 69, 70^. Ranjit *et al*.^47^ demonstrated the separation between collagen I and III based on the use of a phasor plot approach for bone marrow of mice with fibrosis. Different types of collagen production were also studied by means of time-resolved spectroscopy^46^, where the signal was collected from a cell culture producing ECM.

Considering the facts that collagen III is the most abundant collagen in the papillary dermis after collagen I and the connective tissue enriched with collagen III is more flexible^1, 71^, it can be suggested that collagen III is the main ECM component responsible for the lifetime distribution in the inner area of dermal papillae presented in Fig. 5. As the inner area contains the blood vessel, it could be expected that the surrounding tissue is more “soft” compared to the outer area, which is rich in collagen I and is responsible for biomechanical properties of the whole skin. Indeed, while the outer area of dermal papillae exhibits strong SHG signal, no SHG is observed from their inner areas containing blood vessels^13, 40, 42, 72^. Of note, it was demonstrated that the third harmonic generation could be used to visualize the capillary in the inner area of papillae, making possible *in vivo* assessment of blood flow^13^. The geometrical parameters of the capillary-containing inner area of dermal papillae were also used to characterize skin aging *in vivo* using harmonic generation microscopy^43^. The comparison between collagen I and IV distribution in a section of fibrotic mouse kidney clearly demonstrated colocalization of SHG with collagen I, while the inner area containing aorta was filled with collagen IV, which lacks SHG^40^. Collagen IV is known to be the major protein constituent of blood vessel walls, however, the superficial dermal capillary wall thickness is much lower compared to aorta^73^, where even the collagen IV ring was ~50 μm thick. Hence, we consider the hypothesis that the major ECM component of the inner area in the dermal papillae is collagen IV unlikely, however, further histological investigation is necessary to confirm its origin.

## Materials and Methods

### Research objects

*In vivo* measurements were performed on six healthy volunteers on their fingernail bed and inner forearm areas. In total, over 100 images of papillary structures were obtained. The volunteers were instructed not to use any cosmetic products for at least 48 hours and not to take a bath or shower for at least 4 hours previous to the beginning of the measurements. The skin areas selected for the measurements were without hairs, wrinkles and visible distortions or abnormalities. Blood sample taken from a healthy individual was centrifuged for 10 minutes at 1500 RPM (Hettich Zentrifugen, Universal 320R) to obtain blood plasma. RBC measurements were performed in a 0.1 M NaCl solution.

All the experimental protocols and subject recruitment were approved by the Ethics Committee of the Charité-Universitätsmedizin Berlin. Informed consent was obtained from all subjects. All methods were carried out in accordance with relevant guidelines and regulations.

### Confocal laser scanning microscopy (CLSM)

*In vivo* investigations were carried out using a CLSM (VivaScope^®^ 1500 Multilaser, Mavig, Germany) in both fluorescence (excitation wavelength 488 nm) and reflectance (excitation wavelength 785 nm) modes. The utilized CLSM system was described in detail elsewhere^54^. All the images were built using the ImageJ software^74^.

### Multiphoton tomography and fluorescence lifetime imaging

Two-photon *in vivo* imaging was performed with a Dermainspect (JenLab GmbH, Jena, Germany) device equipped with a tunable femtosecond Ti:sapphire laser (Mai Tai XF, Spectra Physics, USA). The laser was operated at 760 nm and generated 100-fs pulses at a repetition rate of 80 MHz. The 410–680 nm bandpass filter was used to detect autofluorescence.

FLIM images were processed in the SPCImage software (Becker&Hickl, Berlin, Germany) incorporated into the Dermainspect system. Fluorescence decay in each pixel was fitted with a sum of two exponentials (fast and slow) with a fixed shift value, and the intensity threshold was chosen depending on the image quality. The obtained lifetime (*τ*_1_ and *τ*_2_) and amplitude (*a*_1_ and *a*_2_) values were further exported and used for the evaluation of lifetime distributions and image segmentation. The average lifetime was defined as *τ*_*m*_ = (*a*_*1*_ *τ*_*1*_ + *a*_*2*_ *τ*_*2*_)/(*a*_*1*_ + *a*_*2*_). All the images were built using the ImageJ software. The utilized MPT-FLIM system has been previously presented in details elsewhere^75^.

## Electronic supplementary material

Video 3

Video 2

Video 1
