# Supplementary figures and images for: Two-photon autofluorescence lifetime imaging of human skin papillary dermis in vivo: assessment of blood capillaries and structural proteins localization
Source: Sci Rep. 2017 Apr 26;7:1171. doi: 10.1038/s41598-017-01238-w (PMC5430894; doi:10.1038/s41598-017-01238-w)

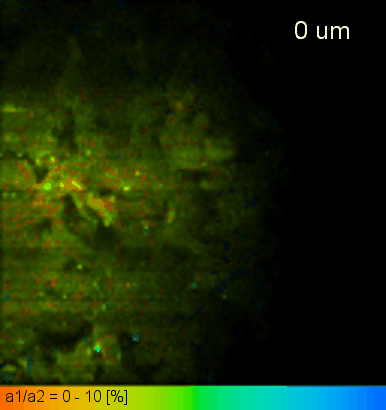

Supplement: Supplementary file 1 — Video 3 [file 41598_2017_1238_MOESM1_ESM.gif]

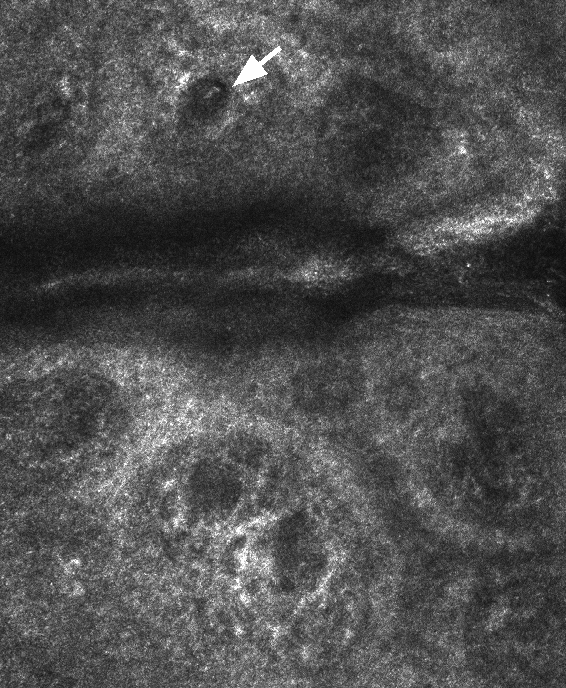

Supplement: Supplementary file 2 — Video 2 [file 41598_2017_1238_MOESM2_ESM.gif]
